# A Strategy for the Proliferation of *Ulva prolifera*, Main Causative Species of Green Tides, with Formation of Sporangia by Fragmentation

**DOI:** 10.1371/journal.pone.0008571

**Published:** 2010-01-05

**Authors:** Shan Gao, Xiaoyuan Chen, Qianqian Yi, Guangce Wang, Guanghua Pan, Apeng Lin, Guang Peng

**Affiliations:** 1 College of Marine Science and Engineering, Tianjin University of Science and Technology, Tianjin, China; 2 Institute of Oceanology, Chinese Academy of Sciences, Qingdao, China; 3 Graduate School, Chinese Academy of Sciences, Beijing, China; Purdue University, United States of America

## Abstract

*Ulva prolifera*, a common green seaweed, is one of the causative species of green tides that occurred frequently along the shores of Qingdao in 2008 and had detrimental effects on the preparations for the 2008 Beijing Olympic Games sailing competition, since more than 30 percent of the area of the games was invaded. In view of the rapid accumulation of the vast biomass of floating *U. prolifera* in green tides, we investigated the formation of sporangia in disks of different diameters excised from *U. prolifera*, changes of the photosynthetic properties of cells during sporangia formation, and development of spores. The results suggested that disks less than 1.00 mm in diameter were optimal for the formation of sporangia, but there was a small amount of spore release in these. The highest percentage of area of spore release occurred in disks that were 2.50 mm in diameter. In contrast, sporangia were formed only at the cut edges of larger disks (3.00 mm, 3.50 mm, and 4.00 mm in diameter). Additionally, the majority of spores liberated from the disks appeared vigorous and developed successfully into new individuals. These results implied that fragments of the appropriate size from the *U. prolifera* thalli broken by a variety of factors via producing spores gave rise to the rapid proliferation of the seaweed under field conditions, which may be one of the most important factors to the rapid accumulation of the vast biomass of *U. prolifera* in the green tide that occurred in Qingdao, 2008.

## Introduction

Green tides are caused by very large accumulations of green macro-algae that occur under suitable conditions, in particular eutrophication [1]–[7]. In the summer of 2008, a large-scale green tide occurred in the Yellow Sea, especially along the shores of Qingdao (35°35′–37°09′N, 119°30′–121°00′E), China [8]. The results of satellite remote sensing monitoring showed that this green tide covered approximately 3800 km^2^ [9]. It was speculated that around 20 million wet tonnes of the biomass of the green macro-algae was produced along the shores of Qingdao, and at least 1.5 million wet tonnes was salvaged (unpublished results). This green tide had a negative impact on recreational beaches, and it invaded more than 30% of the area of the 2008 Beijing Olympic Games sailing competition in Qingdao, seriously hindering the preparations for the Games [9]. Increasing attention has been paid to the event by the government of China and by many people all over the world.

The green tide that occurred along the shoreline of Qingdao consisted predominantly of free-floating thalli of *Enteromorpha* sp. or *Ulva* sp. Some taxonomists have identified the main species in this green tide as *Enteromorpha prolifera* (O.F. Müeller) J. Agardh [10]. It is difficult to distinguish between *Enteromorpha* and *Ulva* species, and there is still some controversy concerning the two genera. According to some scientists *Ulva* and *Enteromorpha* are not distinct genera [11], and therefore *Enteromorpha prolifera* should be known as *Ulva prolifera*. Here, adopting the advice of Hayden *et al.* (2003) [11], we refer to *E. prolifera* as *U. prolifera*.

**Figure 1:**
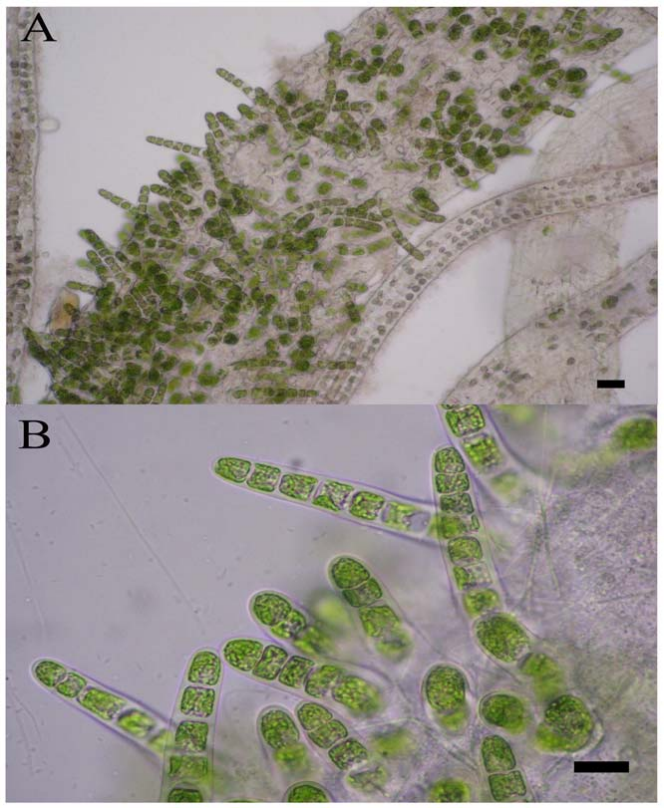
The germination in situ of the spores of *U. prolifera* floating along the shores of Qingdao in the summer of 2008. The scale bars represent 10 µm.

The life-history of *U. prolifera* with a tubular, generally profuse branching and filamentous form consists of similar haploid and diploid phases; namely, gametophyte and sporophyte generation [12]. This seaweed is distributed widely in the intertidal zones of shores and estuaries around the world by virtue of its tolerance of a wide range of salinity and water temperature [13], [14]. It has been reported that the early germination of spores of *Enteromorpha* sp. requires attachment to a solid substratum, such as small sand particles and the thalli [1], and can then grow without the need for attachment to the substratum [15]. There are countless small floating sand particles in the Yellow Sea that could serve as a solid substratum. In addition, according to our oceanographic survey, the spores became attached to the floating thalli and then germinated, i.e. germination *in situ* (Fig. 1A and B), which is in accord with the results reported by Lin *et al*. (2008) [16]; thus, the new individuals were floating.

**Figure 2:**
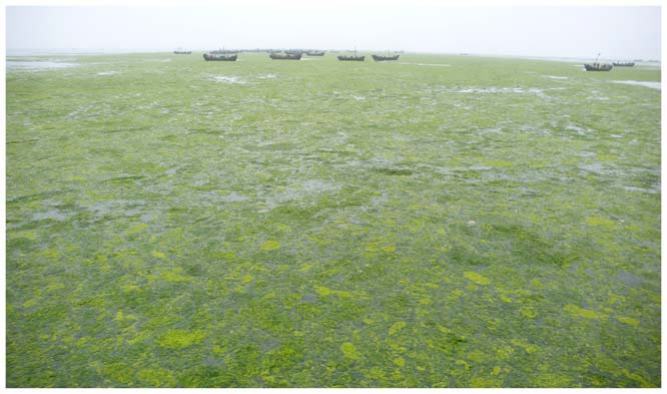
The green tide that occurred along the shores of Qingdao in the summer of 2008.

Due to the detrimental effects on the ecology, especially the coastal ecology [4], [5], [17], [18], green tides have been the focus of many studies, and most have been focused on the causative species—*U. prolifera* [8], [10], [16] and other relevant species [15], [19], [20]. The proliferation of the floating thalli was extremely rapid. For instance, the vast biomass of *U. prolifera* was accumulated along the shores of Qingdao in less than two weeks during the summer of 2008 (Fig. 2). Earlier [16], we reported seven different methods of reproduction of *U. prolifera*; nevertheless, it is still difficult to explain how the biomass accumulated so rapidly. Santeliees and Paya (1989) [21] reported that Chlorophyta fragments caused by grazers or in their excreta could reproduce new individuals. Under field conditions, many other factors, such as waves and propellers, can give rise to the formation of fragments of thalli, and many fragments in the initial phase of green tides in the Yellow Sea were found at the time of our oceanographic survey. Thus, we deduce that there may be a close relationship between the fragments and the rapid accumulation of the vast biomass of the green seaweed. However, little information is available about the process by which the fragments give rise to new individuals. In the present study, we used disks with different diameters excised from *U. prolifera* to investigate the changes of the fragments and the early development of this seaweed, with particular attention to the relationship between the size of the disks and the area of sporangia and spore release.

## Materials and Methods

### Sample Preparation and Culture Conditions

Samples of *U. prolifera* were collected from the coast of Qingdao (35°35′–37°09′N, 119°30′–121°00′E), Shandong Province, China. The thalli were rinsed gently in sterile seawater to remove any sediment, small grazers or epiphytes. The thalli were cultured in sterile seawater (salinity 30psu) at 8°C with 20–30µmol photon m^−2^ s^−1^ using a 12 hours light/12 hours dark cycle for acclimation before the start of experiments.

After recovery (approximately 4 hours), the thalli were cleaned thoroughly with a calligraphy brush under a magnifier. Disks of different diameters were excised from the vegetative thalli with similar health and physiology conditions using a Stiletto apparatus (Harris Uni-core, USA). The disks were 0.50 mm, 0.75 mm, 1.00 mm, 2.00 mm, 2.50 mm, 3.00 mm, 3.50 mm, and 4.00 mm in diameter. In each group, 10 disks were chosen and transferred to a Petri dish containing a slide for adhesion of spores. The Petri dishes were filled with sterile seawater (salinity 30psu) containing nitrogen (500µmol/L) and phosphorus (50µmol/L), and germanium dioxide (2mg/L) to suppress the growth of diatoms. The Petri dishes were incubated at 20°C at an irradiance of 60–90 µmol photon m^−2^ s^−1^ under white light with a 12 hours light/12 hours dark cycle. The culture medium was replaced every two days.

### Microscopic Observations and Determination of Photosynthetic Parameters

Microscopic observations of changes in the cells of the disks were made regularly. When the area of sporangia in the disks was constant and spores were no longer discharged, both the percentages of area of sporangia and spore release in the excised disks were calculated and averaged for the ten disks in each group.

In order to verify the activity of spores and investigate the early development of *U. prolifera*, the slides in the Petri dishes were observed at regular intervals to determine whether the spores were attached. Slides with spores attached were transferred to new Petri dishes containing sterile seawater and nutrients as described above and cultured under the conditions described by Sousa *et al.* (2007) [20]. Subsequently, the focus on the daily observations under a microscope was transferred to the development of spores. The processes, including the formation of sporangia and the development of spores, were recorded with a differential interference contrast microscope (Leica DM2500, Germany).

In the present work, during the formation of sporangia and the early development of *U. prolifera*, the chlorophyll fluorescence of photosystem II (PS II) was determined using pulse amplitude modulation fluorimetry (IMAGING-PAM, Waltz GmbH, Effeltrich, Germany). The photosynthetic parameters were calculated on the basis of the chlorophyll fluorescence. After 5–10 minutes dark-adaptation of the disks, the intrinsic fluorescence (F_0_) from the antenna system of fully oxidized PS II was measured. Then a saturating flash was applied to detect the maximal fluorescence (F_m_) from the fully reduced PS II reaction centers [22]. The variable fluorescence F_v_ was obtained as the difference between F_m_ and F_0_, together with the optimum PS II quantum yield (F_v_/F_m_) [23]. The effective PS II quantum yield (Y (II)) was calculated according to the formula [24]:
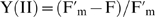



When the disks were illuminated, the maximum fluorescence yield (F′_m_) was detected, which was normally lower than F_m_ due to non-photochemical quenching (i.e. heat dissipation). The current fluorescence, F_t_, was averaged for 3 s, and this value was designated F. The above-mentioned parameters could be read directly in the report window of a computer connected to IMAGING-PAM. Mean values and standard deviations were calculated. All the results in this study were expressed as mean values, and these were used for statistical analysis via ANOVA using the STATISTICA 7.0 software. For post-hoc analysis, the Tukey test was used at α = 0.05 significance level.

## Results

### The Process of Sporulation

**Figure 3:**
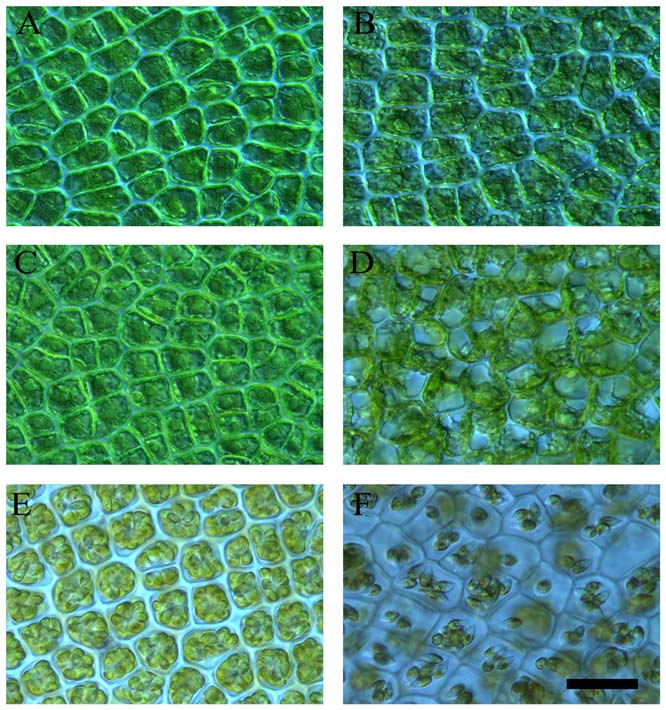
The phases of the formation of sporangia in excised disks from *U. prolifera*. (A) newly excised disks; (B) chloroplasts dispersed; (C) chloroplasts aggregating in the center of cells; (D) vacuoles swollen; (E) spores within the sporangia; (F) partial spores released from sporangia. The scale bars represent 10 µm.

Great changes in the cells of the disks occurred after the excised disks were cultured for some time (Fig. 3). Fig. 3B shows that the chloroplasts were characterized as granular forms as compared with newly excised disks (Fig. 3A). Next, the granular chloroplasts were concentrated in the center of the cells (Fig. 3C). Several hours later, the vacuoles swelled, giving rise to aggregation of the chloroplasts in the cells (Fig. 3D). After about 72 hours, pyriform spores were formed within the sporangia (Fig. 3E) and, subsequently, spores were liberated from the sporangia (Fig. 3F).

**Figure 4:**
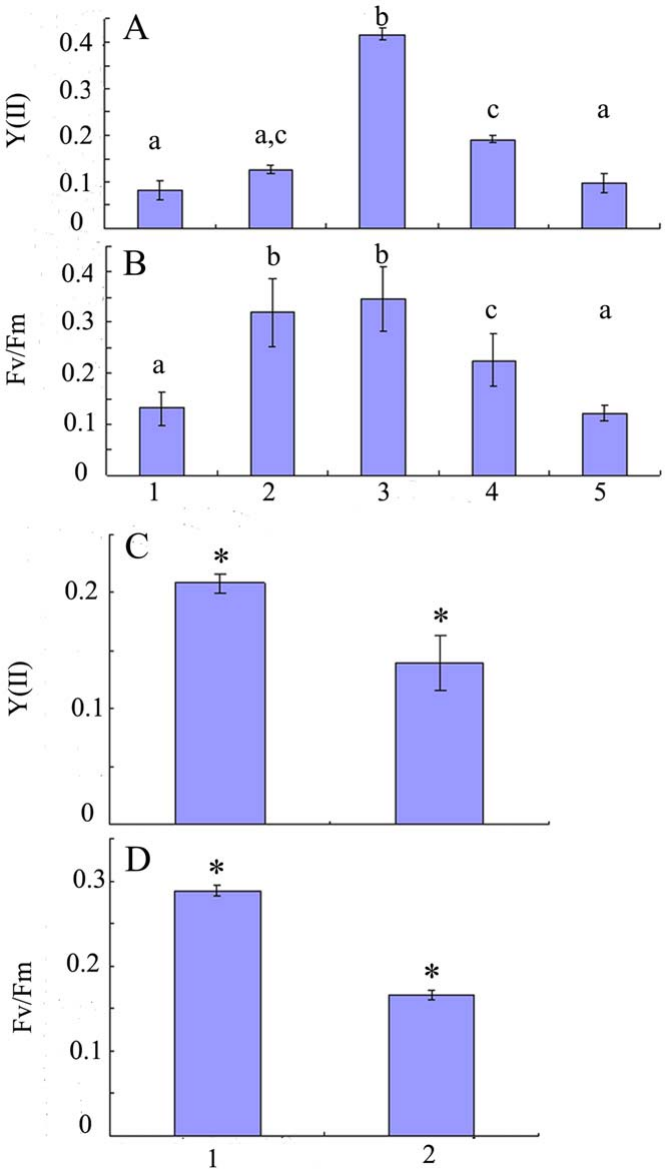
The values of (A) the effective PS II quantum yield [Y (II)] and (B) the optimum PS II quantum yield [F_v_/F_m_] of each phase of the formation of sporangia. 1, Newly excised disks; 2, chloroplast dispersed; 3, chloroplasts aggregated; 4, vacuoles swollen; 5, pyriform spores within sporangia. The values of (C) Y (II) and (D) F_v_/F_m_ of the vegetative cells (1) and that of the mature sporangia (2) in the same excised disks. In (A) and (B), different letters (a, b, c) represent significant differences between the phases of the formation of sporangia (p<0.05, ANOVA, followed by Tukey test for post-hoc comparisons). In (C) and (D), asterisks represent significant differences between the vegetative cells and the mature sporangia (p<0.05, two-sample *t*-test).

Variable tendencies of the photosynthetic parameters involving the effective PS II quantum yield [Y (II)] and the optimum PS II quantum yield [Fv/Fm] during the formation of sporangia were observed (Fig. 4). Clearly, a Y (II) close to 0.1 of cells in the newly excised disks (Fig. 3A) was low, but then rose sharply to the highest values (around 0.4) as the chloroplasts aggregated in the center of cells (Fig. 3C). Subsequently, the yields dropped gradually to about 0.1 as spores were formed (Fig. 3E). In addition, it was apparent that variations of F_v_/F_m_ were similar to those of Y (II). F_v_/F_m_ of each phase were slightly higher than those of Y (II), with the exception that the chloroplasts were concentrated in the cells (Fig. 3C). Furthermore, F_v_/F_m_ increased dramatically from the initial phase to the state where the chloroplasts were dispersed in cells (Fig. 4B). F_v_/F_m_ dropped gradually to the lowest level when spores had been formed (Fig. 4B). As shown in Fig. 4, Y (II) (Fig. 4C) and F_v_/F_m_ (Fig. 4D) of the vegetative cells were both higher than those of the mature sporangia in the same disks. Moreover, there were significant differences between Y (II) and F_v_/F_m_ of the vegetative cells and the mature sporangia (p<0.05; two-sample *t* test).

### The Relationship between the Size of Excised Disks and the Percentage of Area of Sporangia

**Figure 5:**
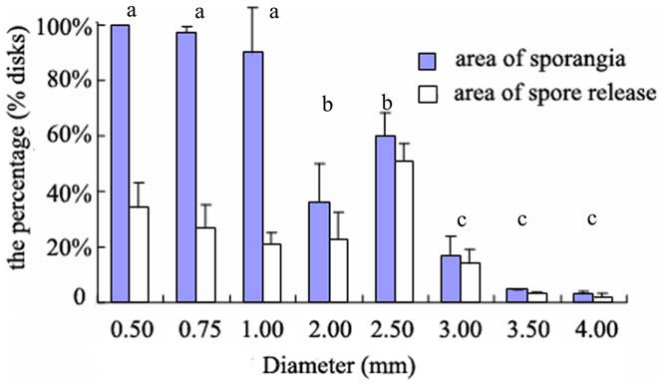
The percentage of area of sporangia and that of spore release to the whole disks. Different letters (a, b, c) represent significant differences between the sizes of disks treatments (p<0.05, ANOVA, followed by Tukey test for post-hoc comparisons).

The number of wounded cells at the marginal of excised disks and the ratio of number of wounded cells to total cells, together with the area of sporangia in different disks are presented in Table 1. Moreover, the percentage of area of sporangia in different disks is shown in Fig. 5. Clearly, there was a significantly different (p<0.05) percentage of the area of sporangia in disks with different diameters (Fig. 5). After the excised disks were cultured for about 96 hours, the disks of 0.50 mm and 0.75 mm diameter, in which the ratio of number of wounded cells to total cells was much higher than others (Table 1), had transformed almost completely into sporangia; in other words, the percentage of area of sporangia was close to 100%. The value was >90% for the 1.00 mm diameter disks, and close to 40% and 60% for the 2.00 mm and 2.50 mm disks, respectively. There were only significant differences between the disks ≤1.00 mm and the 2.00 mm and 2.50 mm disks (p<0.05; Tukey test). In contrast, the value was <15% in the 3.00 mm, 3.50 mm, and 4.00 mm diameter disks in which the ratio (about 1%) of number of wounded cells to total cells was lower than that in the smaller disks ≤1.00 mm. Statistically significant differences occurred between the value in 2.00 mm and 2.50 mm diameter disks and the value in disks ≥3.00 mm (p<0.05; Tukey test). It appeared that sporangia were present only at the marginal and submarginal cells of the disks ≥3.00 mm in diameter.

**Table 1:** The number of wounded cells, the ratio of wounded cells to total cells and the area of sporangia and spore release in disks with different diameters.

Disks(mm)	Number of wounded cells	Total cells	Wounded cells/total cells (%)	Area of sporangia (mm^2^)	Area of spore release (mm^2^)
**0.50**	157±12	1973±128	8%	0.1963±0	**0.0706±0.0054**
**0.75**	236±17	4426±205	5.3%	0.4253±0.0070	**0.1063±0.0018**
**1.00**	320±15	7951±189	4%	0.7584±0.0136	**0.1669±0.0030**
**2.00**	628±22	32315±247	2%	0.9606±0.1499	**0.6436±0.1004**
**2.50**	785±18	49073±251	1.6%	3.1155±0.1094	**2.5547±0.0897**
**3.00**	942±25	70650±195	1.3%	1.6104±0.2158	**1.1273±0.1511**
**3.50**	1099±23	96193±305	1.1%	0.5520±0.0773	**0.3312±0.0464**
**4.00**	**1266±27**	**137510±352**	**0.92%**	**0.1851±0.0347**	**0.0925±0.0173**

### The Relationship between the Size of Excised Disks and the Percentage of Area of Spore Release

Fig. 5 shows the percentage of area of spore release in disks with different diameters. The area of spore release in different disks is presented in Table 1. The percentage of area of spore release to sporangia in the 0.50 mm, 0.75 mm, and 1.00 mm diameter disks was <36%. The highest percentage of area of sporangia was observed in the 0.50 mm and 0.75 mm diameter disks, yet the highest percentage of area of spore release did not occur in these disks. Unexpectedly, it was in the 2.00 mm and 2.50 mm diameter disks that the largest percentage (about 82%) occurred. In contrast, the percentage of area of spore release was <70% in the 3.00 mm, 3.50 mm, and 4.00 mm diameter disks. There were significant differences between the disks ≤1.00 mm and the ones of 2.00 mm and 2.50 mm diameter (p<0.05; Tukey test). The differences of the percentage of area of spore release between the disks of 2.00 mm and 2.50 mm diameter and the ones ≥3.00 mm were also significant (p<0.05; Tukey test). Additionally, the release of spores was first observed in the excised 2.00 mm and 2.50 mm diameter disks.

### The Development of Spores

**Figure 6:**
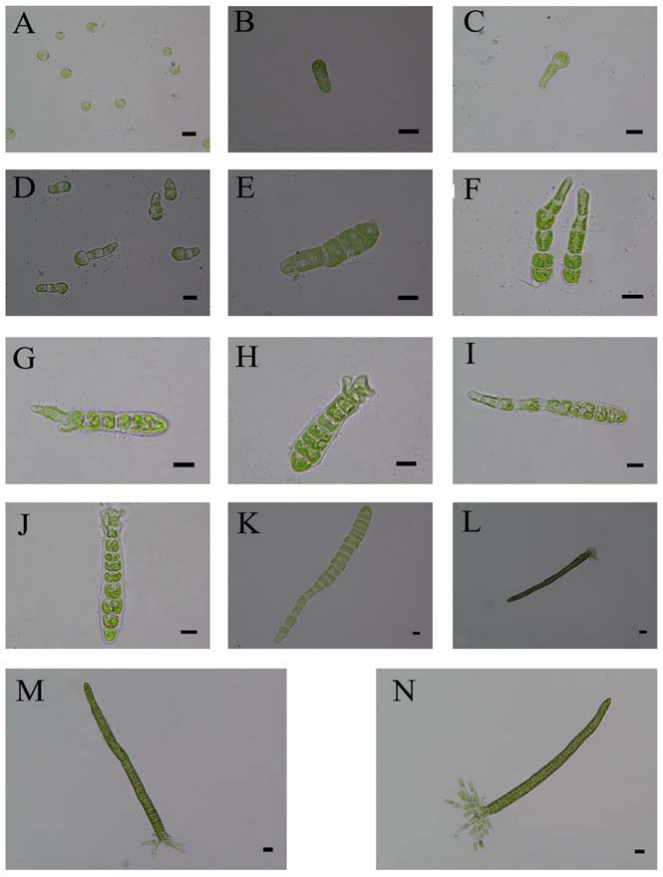
The phases of the early development of *U. prolifera*. The scale bars represent (A–J) 10 µm and (K–N) 20 µm.

**Figure 7:**
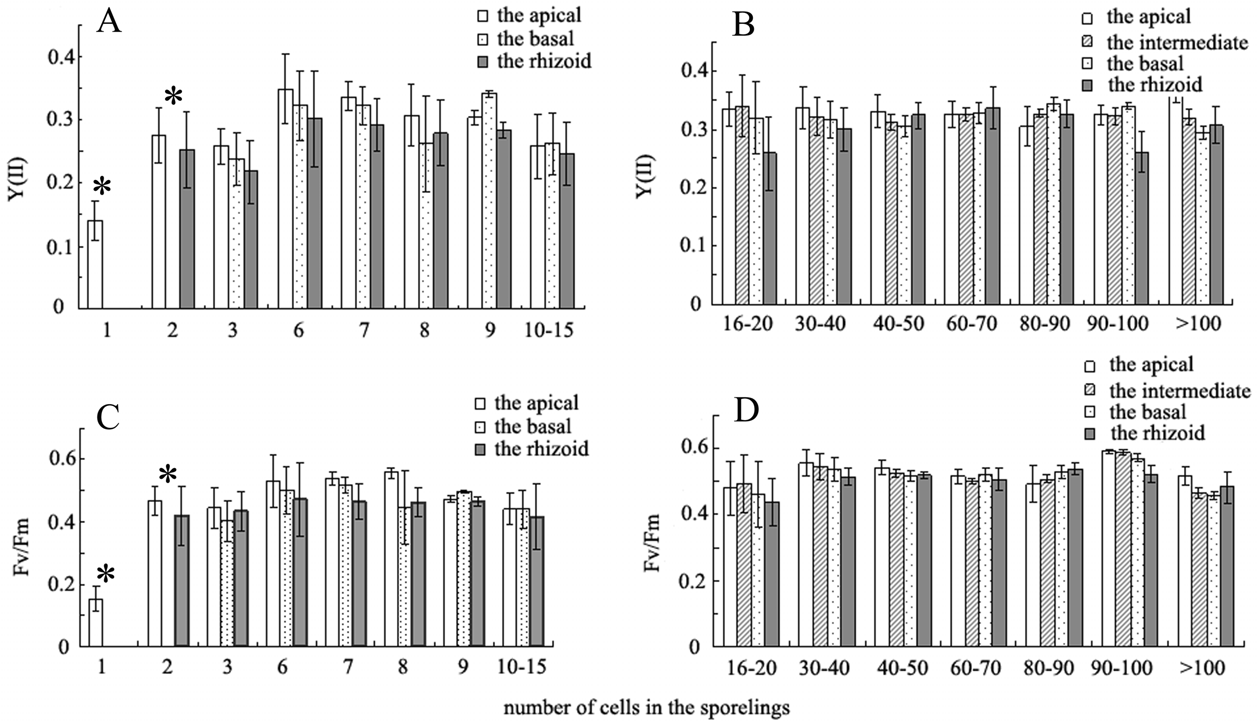
The values of Y (II) and F_v_/F_m_ of the phases of the development of *U. prolifera*. Asterisks represent significant differences between the apical and the rhizoid in the same sporeling (p<0.05, two-sample *t*-test).

Fig. 6 shows that the spores discharged from the excised disks developed into new individuals. The pyriform spores newly discharged from sporangia were soon attached to the slides and then germinated (Fig. 6B). Subsequently, the settled spores started to divide (Fig. 6C and D). Several hours later, the basal cells increased in length and the apical cells underwent division (Fig. 6E–K). With the number of cells increasing, the rhizoid and the linear thalli were formed (Fig. 6L–N). On the other hand, there were large changes of the photosynthetic properties of the sporelings during the early development of *U. prolifera* (Fig. 7). The Y (II) and F_v_/F_m_ values of the settled spores were close to 0.15, and were lower than those of the other phases (Fig. 7). When spores divided into two cells (Fig. 6C), the Y (II) and F_v_/F_m_ values of the apical cell were around 0.28 and 0.5, respectively, which was significantly higher (p<0.05) than those of the basal cell (about 0.25 and 0.4). The Y (II) and F_v_/F_m_ of the apical cell were always higher than those of the basal cell in sporelings with several cells (Fig. 7A and C). With the number of cells increasing, the differences of Y (II) and F_v_/F_m_ among the apical, the intermediate, the basal and the rhizoid cells were not significant (Fig. 7B and D).

## Discussion

Pulse amplitude-modulated chlorophyll fluorescence has the advantages that it is convenient, nonintrusive and rapid and has been a useful tool for assessing the macro-algal physiological state under different conditions [22], [25], [26]. The technique is widely used for measuring the influence of environmental stress on the physiological state of aquatic photosynthetic organisms [26], and it is attractive for making qualitative and even quantitative comparisons of photosynthetic properties of cells during differentiation and development. On the basis of the in vivo chlorophyll fluorescence yields of PS II determined by PAM fluorimetry, the photosynthetic parameters are calculated with established formulae [24], [27]. The most useful is Y (II), which represents the proportion of the light absorbed by chlorophyll in PS II that is used in photochemistry. F_v_/F_m_, another widely used parameter, provides information on the potential quantum efficiency of PS II, and is used as a sensitive indicator of photosynthetic performance of photosynthetic organisms [28]. The application of PAM fluorimetry in the formation of sporangia and the development of *U. prolifera* spores will help us to understand these processes.

According to our results, the effect of the size of the excised disks on the percentage of area of sporangia was significant (p<0.05). The ratio of number of wounded cells to total cells in the smaller disks ≤1.00 mm diameter was higher than that of the larger disks (≥3.00 mm diameter). The percentage of area of sporangia in the smaller disks, with diameters such as 0.50 mm, 0.75 mm and 1.00 mm, was much higher than that of the larger disks (≥3.00 mm diameter) (Fig. 5). In these larger disks, the sporangia were formed only at the marginal and submarginal cells, which was similar to the calli in higher plants that are generated only at the site of wounding [29], [30]. All these suggested that the higher ratio of wounded cells to total cells in excised disks may promote significantly the formation of sporangia. These results were similar to those reported by Dan *et al.* (2002) and Hiraoka and Oka (2008) [31], [32]. On the other hand, although the percentage of sporangia formed in disks with ≤1.00 mm diameter were close to 100%, the largest ratio of area of spore release to that of sporangia occurred in the disks with 2.00 mm and 2.50 mm diameter covering certain vegetative cells (Fig. 5 and Table 1). To summarize, the formation of sporangia was affected significantly by the size of the excised disks, and the smaller disks were more conducive to forming sporangia than the larger ones. Additionally, there was a close relationship between spore release and the vegetative cells in disks.

Fig. 4 shows the variation of the photosynthetic parameters, including the effective PS II quantum yield [Y (II)] and the optimum PS II quantum yield [F_v_/F_m_], which fluctuated markedly during sporangia formation. Both increased sharply to the highest level before the sporangia were formed, and subsequently dropped to lower values after sporangia were formed. This demonstrated there was an accumulation of numerous photosynthetic products, such as carbohydrates, to prepare for the formation of sporangia. In addition, as shown in Fig. 4C and D, the Y (II) and F_v_/F_m_ of the mature sporangia were both much lower than those of the vegetative cells (p<0.05). Moreover, as mentioned above, there was a smaller percentage of area of spore release in disks without vegetative cells. We suggest there is a possibility that some substances necessary for spore release were supplied to the sporangia by the adjacent vegetative cells and transported through the plasmodesmata as described by Lobban and Wynne (1981) [33].

During the early development of *U. prolifera*, the Y (II) and F_v_/F_m_ of the apical cells were both higher (p<0.05) than those of the basal ones, suggesting that the photosynthetic properties of the two parts were significantly different. Wang *et al.* (2006) [34] reported that the morphological characteristics of the apical cells were different from those of the basal ones. Thus, our physiological data together with the results described by Wang *et al.* (2006) [34] demonstrated that the early development of *U. prolifera* had polarity. With the number of cells increasing (Fig. 6F–N), all the photosynthetic properties of different regions increased, which included the apical, the intermediate, the basal and the rhizoid cells in sporelings, and the differences among them were not significant (p>0.05). Overall, the results suggested the spores, which were released from the sporangia formed in the excised disks, were vigorous, germinated normally, and developed successfully into new individuals (Fig. 6).

Both eutrophication and climatic conditions such as appropriate light and temperature conditions favor the proliferation and growth of causative species of green tides such as *Ulva.* sp [7], [35]. Consequently, these factors may contribute to the occurrence of green tides [35], which may play important roles in the proliferation and growth of *U. prolifera* in the green tide occurred along the shores of Qingdao, 2008. However, the aforementioned factors are not sufficient to explain the rapid proliferation of *U. prolifera* in the green tide. According to our oceanographic survey, we found that there were numerous fragments with different size in seawater at the initial phase of the green tide occurred in Qingdao, 2008. Actually, under field conditions, due to a variety of factors, such as the actions of grazers [21], waves and propellers, the formation of fragments is inevitable. Thus, we investigated the disks (fragments) of different sizes excised from *U. prolifera* in the present study. The results indicated that the fragments of the appropriate size can produce a large number of spores able to develop successfully into new individuals. Therefore, in spite of many ways of reproduction of *U. prolifera* such as sexual reproduction and vegetative propagation under field conditions [16], we believe that the fragments via producing spores play a crucial role in the rapid accumulation of a vast biomass of *U. prolifera* and may be one of the most important factors which hasten the occurrence of the green tide along the shores of Qingdao, 2008.
